# The effect of a community-based group intervention on chronic disease self-management in a vulnerable population

**DOI:** 10.3389/fpubh.2023.1221675

**Published:** 2023-08-21

**Authors:** Sophie A. Korenhof, Ellen V. Rouwet, Liset E. M. Elstgeest, Irene N. Fierloos, Siok Swan Tan, Marta M. Pisano-Gonzalez, An L. D. Boone, Yves-Marie Pers, Alberto Pilotto, Mónica López-Ventoso, Isabel Diez Valcarce, Xuxi Zhang, EFFICHRONIC Consortium, Hein Raat

**Affiliations:** ^1^Department of Public Health, Erasmus University Medical Center, Rotterdam, Netherlands; ^2^Reinier Academy, Reinier de Graaf Hospital, Delft, Netherlands; ^3^Research Group City Dynamics, InHolland University of Applied Sciences, Rotterdam, Netherlands; ^4^Research Group “Community Health and Active Aging” of the Research Institute of Asturias (IPSA), General Directorate of Care, Ministry of Health, Oviedo, Spain; ^5^Public Health General Directorate, Ministry of Health of the Principality of Asturias, Oviedo, Spain; ^6^IRMB, University of Montpellier, INSERM, CHU Montpellier, Montpellier, France; ^7^Clinical Immunology and Osteoarticular Diseases Therapeutic Unit, Department of Rheumatology, Lapeyronie University Hospital, Montpellier, France; ^8^Department of Geriatric Care, Orthogeriatrics and Rehabilitation, Galliera Hospital, Genoa, Italy; ^9^Department of Interdisciplinary Medicine, University of Bari, Bari, Italy; ^10^Department of Social Medicine and Health Education, School of Public Health, Peking University, Beijing, China

**Keywords:** chronic disease management, self-management, risk factors, vulnerable populations, socioeconomic factors, caregivers

## Abstract

**Introduction:**

Chronic non-communicable diseases (NCDs) are predominantly related to modifiable health behaviors and account for 74% of global deaths at present. Behavior modification through self-management is a strategy to prevent NCDs. Chronic Disease Self-Management Programs (CDSMPs) have demonstrated improvements in health behaviors, health status, and use of healthcare.

**Objective:**

We evaluated the effects of a 6-week CDSMP on self-efficacy, health behaviors, mental health, health-related quality of life (HR-QoL), and health responsibilities among vulnerable populations with chronic disease in Europe.

**Methods:**

A prospective cohort study with a 6-month pre-post single-group design was conducted in five European countries. The intervention targeted adults with chronic conditions and low socioeconomic status, as well as their caregivers. The intervention was a 6-week community-based CDSMP in a group setting. Outcomes were measured per self-report questionnaire at baseline and 6-month follow-up: self-efficacy, health behaviors, mental health, HR-QoL, and health responsibilities.

**Results:**

Of 1,844 participants, 1,248 (67.7%) completed follow-up and attended ≥4 sessions. For the chronic condition group, the following outcome measures at follow-up significantly improved compared with baseline (all *P* < 0.002): self-efficacy (SEMCD-6 6.7 vs. 6.4), mental health (PHQ-8 6.3 vs. 7.0), HR-QoL (SF-12 PCS 42.3 vs. 40.2, SF-12 MCS 42.8 vs. 41.4), health utility (EQ-5D-5L 0.88 vs. 0.86), self-rated health (EQ-5D-5L 67.2 vs. 63.9), communication with healthcare providers (2.28 vs. 2.11), understanding information (3.10 vs. 3.02), number of doctor visits (3.61 vs. 4.97), accident and emergency department visits (0.25 vs. 0.48), total nights in a hospital (0.65 vs. 1.13), and perceived medical errors (19.6 vs. 28.7%). No significant changes were detected in dietary habits, physical activity, substance use, and sleep and fatigue. For caregivers without a chronic condition, only doctor visits significantly decreased (1.54 vs. 2.25, *P* < 0.001).

**Discussion:**

This CDSMP was associated with improvement in self-efficacy, depression, HR-QoL, and health responsibilities over 6 months in a diverse European population with a chronic condition. However, additional interventions targeting lifestyle risk factors are needed to improve health outcomes.

## Introduction

Chronic non-communicable diseases (NCDs) are currently the most common cause of morbidity and mortality, accounting for 74% of all global deaths (1–3). It is estimated that chronic diseases will cost $47 trillion in gross domestic product from 2011 to 2025 globally (4). The development of the most common NCDs is largely related to modifiable lifestyle factors, including smoking, physical activity, stress, and poor dietary habits (5, 6). Hence, lifestyle-related chronic diseases are currently targeted with measures to manage modifiable risk factors, such as increasing physical activity, improving dietary habits, smoking cessation, and stress management (7, 8). Behavior modification is, thus, a crucial strategy for the prevention and treatment of lifestyle-related chronic diseases (9). Programs that enhance self-management may be useful in improving behavioral risk factors (10, 11). Chronic Disease Self-Management Programs (CDSMPs) have demonstrated significant improvements in health behaviors and health status as well as reduced healthcare utilization (12).

Vulnerable populations carry a higher burden of lifestyle risk factors and lifestyle-related chronic diseases (13–17). A vulnerable population can be defined as those at increased risk for chronic non-communicable diseases and refers to a wide range of groups, such as economically disadvantaged people, along with uninsured, racial, and ethnic minorities, older people, and those who meet barriers when accessing healthcare (18, 19). Especially, currently, with overloaded healthcare systems, it has become increasingly important to lower the burden of chronic diseases and reduce health disparities; thus, CDSMPs could be a potential low-cost solution. The objective of our study was to evaluate the effects of a 6-week CDSMP on self-efficacy, health behaviors, mental health, HR-QoL, and health responsibilities among vulnerable populations in five European regions.

## Methods

### Study design

The evaluation study of the EFFICHRONIC project was a prospective cohort study with a 6-month pre-post single-group design and was conducted between January 2018 and November 2020 in five European countries [the Netherlands, Italy, the United Kingdom (UK), Spain, and France]. The EFFICHRONIC project aimed to evaluate the benefits of a CDSMP in managing and maintaining the health of citizens with a low socioeconomic status (SES) and one or more chronic conditions, as well as their caregivers (20). For full details of the study design and protocol, see Tan et al. (20). There were no major deviations from the published protocol study. ISRCTN registry number is ISRCTN70517103 and the date of registration was 20 June 2018. Participant data were collected before the start of the first workshop session at baseline and 6-month follow-up. Ethical approval was provided by the human research ethics board of the study sites (20). All participants provided written informed consent.

### Recruitment and eligibility criteria

Participants were recruited to participate in a CDSMP intervention in their local study site (Occitanie region in France, Genoa province in Italy, Rotterdam region in the Netherlands, Principality of Asturias in Spain, and a region of London in the UK). Citizens were recruited through clinicians, public events and announcements, local patient or volunteer organizations, and community advocates. Recruitment sites were chosen based on their location in distinct environments. Vulnerability maps were constructed in three study sites (Occitanie region, Genoa province, and Principality of Asturias) based on EUROSTAT's NUTS-3 level geographical areas, in which the prevalence of the target population was high (21). For complete recruitment strategies, see Alvarez Rosete et al. (22).

The intervention targeted a vulnerable population of citizens of ≥18 years and with a low SES with one or more chronic conditions as specified in the International Classification of Primary Care (ICPC-2) present for at least 6 months (23). Caregivers of the participants with a chronic condition were also included. Citizens were only eligible to participate if they were able to comprehend the information provided in the local language and were likely to complete the 6-month study duration. Citizens were not eligible to participate when they were experiencing a crisis period, their basic housing needs were not met, they were diagnosed with severe mental health problems, or they suffered from active addictive disorders or cognitive impairment.

### Intervention

The CDSMP is a six-session (2.5 h weekly) community-based intervention built on the self-efficacy theory (10, 24, 25), a well-established program developed by Stanford University and assessed for over 20 years by the Self-Management Resource Center (24, 26). Each group session was led by trained (public) health professionals and trained laypersons in groups of up to 20 participants in the local language (27). Session leaders were trained by certified trainers, following the Stanford methodology (26). Topics included in the sessions were as follows: an overview of self-management and chronic health conditions, making an action plan, relaxation and management of cognitive symptoms, problem-solving, emotions (anger, fear, and/or frustration), fitness, fatigue management, healthy eating, advance directives, communication, medication, making treatment decisions, depression, informing the healthcare team, and working with healthcare professionals (12). The program focused on problem-solving, decision-making, confidence building, management of emotions, positive health and efficient communication to strengthen self-efficacy (i.e., the confidence in one's ability to accomplish a specific task or reach a goal), and managing different aspects of one's health functioning (25).

### Outcome measures

Outcomes were obtained through self-report questionnaires at the start of the first session (baseline) and 6-month follow-up. Caregivers filled out the questionnaires by themselves but not for the individuals with a chronic condition they cared for. The degree of self-efficacy was measured with the Self-Efficacy for Managing Chronic Diseases 6-item scale (SEMCD-6), with scores ranging from 6 to 60, with higher scores corresponding to higher self-efficacy (28). The following health behaviors were assessed: (1) dietary habits: two items on the intake of fruit and vegetables; (2) physical activity: six items on physical exercise (12) and one item on sedentary behavior: International Physical Activity Questionnaire (IPAQ) (29); (3) substance use: current smoking, yes/no; frequency of alcohol use, one item from the Alcohol Use Disorders Identification Test (AUDIT-C) (30); and (4) sleep and fatigue: visual analog scale (VAS), with scores ranging from 0 to 10, with higher values indicating worse sleep/more fatigue. Depression severity was assessed with the Patient Health Questionnaire 8-item scale (PHQ-8), with scores ranging from 0 to 24, with higher values indicating a higher severity, score ≥10 corresponding to current depression (31). Health-related quality of life (HR-QoL) was assessed with the 12-item Short-Form Health Survey (SF-12), with scores ranging from 0 to 100; the EuroQol-5 Dimensions-5 levels (EQ-5D-5L), using the United Kingdom value sets, with scores ranging from 0 to 1, with higher values indicating better health utility (32, 33); and EQ-VAS, with scores ranging from 0 to 100, with higher values indicating better HR-QoL.

Health responsibilities were assessed as follows: (1) communication with healthcare professionals: three items on preparing a list of questions, asking questions, and discussing personal problems (12); (2) health literacy: two items on the Health Literacy Questionnaire (HLQ) (34); (3) healthcare utilization in the past 6 months was assessed with four items on the number of doctor visits, the number of accident and emergency department visits, number of overnight stays in the hospital, and the total number of nights spent in the hospital; (4) medication adherence: six items from the Simplified Medication Adherence Questionnaire (SMAQ) (35); and (5) perceived medical errors: three items on the understandability of a healthcare professional's explanation and prevalence of perceived medical errors from the American Association of Retired Persons (AARP) “survey beyond 50.09” questionnaire (36).

### Other measures

Sociodemographic characteristics were assessed using self-report questionnaires: age, sex, household composition, educational level, income, migration background, employment situation, housing situation, social relationships, and social support. The participant's household composition, income, housing situation, social relationships, and social support were measured by an adapted version of Gijón's Social-Familial Evaluation Scale (SFES) (37). The subjective improvement in the most important outcomes of the CDSMP intervention experienced by the participants was evaluated with seven items at the 6-month follow-up: change in doing at least one activity for health; not letting health problems control their life; the ability to make decisions; the ability to express themselves; in the way of communication with family, friends, and others; confidence in the health system understanding their needs; and satisfaction with the intervention as a whole.

### Statistical methods

Participant characteristics were described using mean (SD) or number of participants (%) for the total study sample. Participant's sociodemographic characteristics and health outcomes were evaluated at baseline after stratification for chronic disease status. Caregivers with a chronic condition were added to the “chronic condition group,” and caregivers without a chronic condition were added to the “no chronic condition group.” *T*-tests were used to compare the means for continuous variables and Pearson's chi-squared tests for categorical variables. To assess the effects of the intervention on continuous outcome measures, linear regression analyses were conducted with a change in the outcome measure between baseline and follow-up as a dependent variable and the beta of the intercept indicating the effect. For dichotomous outcome measures, paired McNemar's tests were used. Outcome analyses were stratified by chronic disease status. In addition, stratified linear regression analyses were run for country, educational level, sex, and age (age <65 years or ≥65 years). Supplementary Tables 2B–E considering 26 outcome measures, the two-sided significance threshold, after Bonferroni correction for multiple testing, was set at a *P*-value = 0.05/26 = 0.0019. In the protocol study, two outcome measures were considered in five countries. Analyses were conducted with SPSS version 25.0 (IBM SPSS Statistics for Windows, IBM Corp., Armonk, NY, USA).

## Results

### Description of participants

Participants were recruited for the project between January 2018 and March 2020; 2,759 participants who fulfilled the inclusion criteria provided informed consent and started the intervention. Of the 1,693 participants who filled in the baseline questionnaire and had an available chronic disease status, 1,377 (81.3%) were citizens with a chronic condition, and 316 (18.7%) were caregivers. Participants were included in the five European study sites: the Netherlands (*n* = 388), Italy (*n* = 331), the United Kingdom (*n* = 345), Spain (*n* = 568), and France (*n* = 212). Table 1 shows the baseline characteristics of the study sample stratified by chronic disease status, i.e., having a chronic condition or being a caregiver without a chronic condition. Supplementary Table 1A presents the baseline characteristics of the total study population, and Supplementary Table 1B describes the baseline characteristics at the country level. Compared with caregivers without a chronic condition (the no chronic condition group), citizens with a chronic condition were, on average, less often female, reported more current depression, more often lived alone, had a lower educational status, had a lower income, less often had a migration background, and less often worked.

**Table 1:** Baseline characteristics of the study sample stratified by chronic condition status (*n* = 1,693).

	**Chronic condition (*n* = 1,377)**	**No chronic condition (*n* = 316)**	***p*-value**
Age, years	60.2 (14.5)	52.5 (14.6)	0.538^*^
Sex, % female	903 (66.3%)	246 (79.9%)	**<0.001** ^†^
**Study site**			**<0.001** ^†^
The Netherlands	219 (15.9%)	52 (16.5%)	
Italy	238 (17.3%)	93 (29.4%)	
United Kingdom	331 (24.0%)	14 (4.4%)	
Spain	404 (29.3%)	134 (42.4%)	
France	185 (13.4%)	23 (7.3%)	
**Current smoking**	223 (16.5%)	50 (16.2%)	0.889^†^
Alcohol use ≥4 times/week	170 (12.5%)	24 (7.7%)	0.016^†^
Aerobic physical activity <150 min/week	120.3 (104.5)	141.8 (112.1)	0.029^†^
Fruit <3 servings/day	1,122 (82.6%)	267 (84.8%)	0.362^†^
Vegetables <3 servings/day	1,196 (88.3%)	275 (87.6%)	0.735^†^
Current depression (PHQ-8 ≥10)	393 (31.3%)	30 (10.6%)	**<0.001** ^†^
Household composition, % living alone	448 (33.4%)	57 (18.9%)	**<0.001** ^†^
**Education**			**<0.001** ^†^
Primary or no education	279 (20.8%)	39 (12.7%)	
Secondary	820 (61.1%)	190 (62.1%)	
Tertiary or higher	243 (18.1%)	77 (25.2%)	
**Income (netto)**			**<0.001** ^†^
>€2,130 per month	384 (29.7%)	103 (35.0%)	
€1,420–2,130 per month	381 (29.5%)	91 (31.0%)	
€994–1,419 per month	253 (19.6%)	34 (11.6%)	
<€993 per month	100 (7.7%)	51 (17.3%)	
Disability or social benefit	175 (13.5%)	15 (5.1%)	
**Housing adaptation**			0.117^†^
Adapted to my needs	1,099 (82.1%)	262 (87.9%)	
Reduced accessibility	94 (7.0%)	15 (5.0%)	
Not properly equipped	47 (3.5%)	8 (2.7%)	
No elevator or phone	42 (3.1%)	8 (2.7%)	
Declining or uninhabitable	57 (4.3%)	5 (1.7%)	
**Migration background**	218 (16.1%)	76 (24.8%)	**<0.001** ^**†**^
Working status, % not working	420 (33.0%)	168 (57.3%)	**<0.001** ^†^

Data are mean (SD) or number of participants (%).

The chronic condition stratum includes caregivers with a chronic condition.

PHQ-8, Patient Health Questionnaire; SD, standard deviation. Net, Net income is the amount of money a person earns after taxes and deductions are taken out.

* p-value based on t-test; significant p-values in bold.

† p-value based on Pearson's chi-square test; significant p-values in bold.

### Adherence to interventions and follow-up attrition

**Figure 1:**
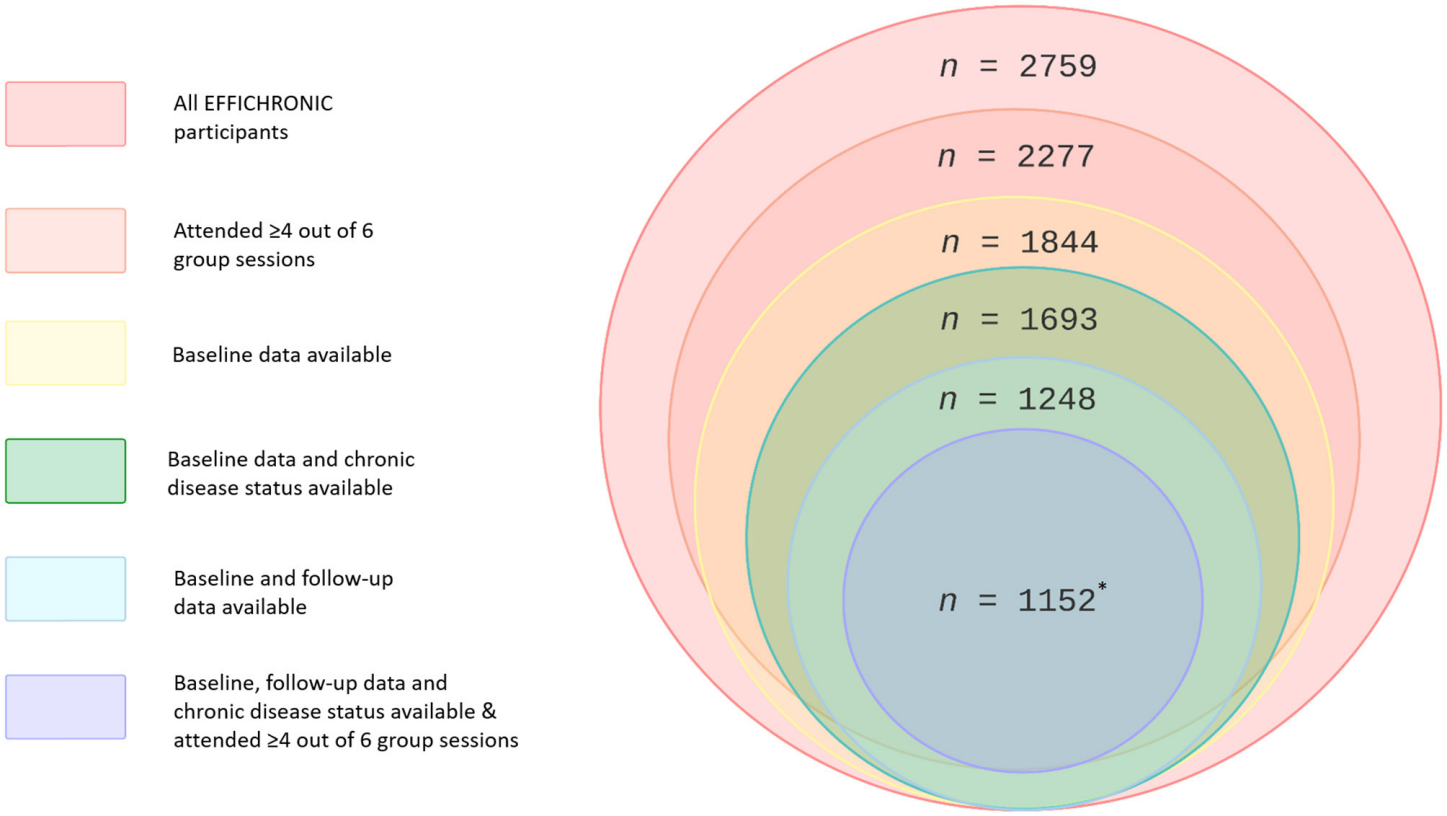
Participant flow chart. *Number per country: Netherlands, *n* = 194; Italy, *n* = 214; United Kingdom, *n* = 275; Spain, *n* = 336; France, *n* = 133.

Of all included participants (*n* = 2,759), 2,277 (82.5%) attended at least four out of six sessions of the CDSMP intervention. Attending ≥4 out of six group sessions was defined as “good adherence” (38). A total of 1,844 participants (66.8% out of 2,759) took part in the baseline questionnaire, and 1,252 participants took part in the follow-up questionnaire. Four participants only took part in the follow-up questionnaire. The number of participants that completed both baseline and follow-up was 1,248. Overall, 1,152 out of 1,844 (62.5%) participants took part in both baseline and 6-month follow-up questionnaires, attended ≥4 sessions, had an available chronic disease status, and were included as the study sample for further analyses. The baseline characteristics of the 692 excluded participants were compared with the included participants. On average, the excluded participants were younger, more often female, more often a current smoker, and spent less time on aerobic physical activity; there were also significant differences in country, education level, income, and housing adaptation (see Supplementary Table 3). A flow chart of the participants is shown in Figure 1.

### Outcomes

Table 2 presents the outcome scores of the 1,152 participants who completed both the baseline and follow-up questionnaire, attended ≥4 sessions, and had an available chronic disease status. Among participants with a chronic condition (*n* = 951), there were significant improvements at follow-up compared with baseline (all *P* < 0.002) in self-efficacy (SEMCD-6 6.7 vs. 6.4), PHQ depression scores (6.3 vs. 7.0), physical and mental HR-QoL (PCS 42.3 vs. 40.2, MCS 42.8 vs. 41.4), health utility (EQ-5D-5L 0.88 vs. 0.86), and self-rated overall health (EQ-5D-5L 67.2 vs. 63.9). In addition, a significant improvement was shown in communication with healthcare providers (2.28 vs. 2.11) and understanding information (3.10 vs. 3.02). Furthermore, within the healthcare utilization, the number of doctor visits (3.61 vs. 4.97), Accident and Emergency department visits (0.25 vs. 0.48), and total nights spent in a hospital (0.65 vs. 1.13), all measured over the last 6 months, significantly diminished. Finally, the percentage of perceived medical errors (19.6 vs. 28.7%) decreased. No significant changes were shown in dietary habits, physical activity, substance use, and sleep and fatigue. In addition, finding health information, the number of overnight hospital visits, medication adherence, perceived unclear communication with the doctor, and perceived experienced medical errors as a problem did not change.

**Table 2:** Effects of the EFFICHRONIC intervention stratified by chronic condition status (*n* = 1,152).

	**Chronic condition (*****n*** = **951)**				**No chronic condition (*****n*** = **201)**			
**Outcomes**	**Baseline**	**Follow-up (6 month)**	**Effect variable**	***p-*** **value** ^#^	**Baseline**	**Follow-up**	**Effect Variable**	***p*** **-value** ^#^
**Self-efficacy**								
SEMCD-6 (range 1–10)^§^	6.4 (2.1)	6.7 (2.0)	0.350	**<0.001** ^*^	8.1 (1.8)	8.2 (1.8)	0.019	0.872^*^
**Health behaviors**								
**Dietary habits**								
Fruit <3 portions/day	766 (82.0%)	763 (81.7%)	0.96	0.872^†^	169 (84.5%)	164 (82.0%)	0.71	0.458^†^
Vegetables, <3 portions/day	817 (88.0%)	801 (86.3%)	0.75	0.156^†^	165 (83.8%)	169 (85.8%)	1.55	0.557^†^
**Physical activity**								
Stretching/strengthening (min/week)	30.7 (51.2)	29.1 (47.6)	−1.033	0.533^*^	39.2 (59.9)	50.5 (64.5)	12.395	0.006^*^
Aerobic physical activity (min/week)	123.3 (103.3)	128.3 (106.9)	5.225	0.104^*^	152.0 (106.0)	148.1 (107.1)	−4.015	0.591^*^
Sedentary behavior (h/day)	5.9 (2.9)	5.7 (2.9)	−0.163	0.062^*^	5.2 (2.9)	4.9 (2.5)	−0.399	0.018^*^
**Substance use**								
Current smoking	129 (14.1%)	128 (14.0%)	0.94	1.000^†^	24 (12.5%)	27 (14.1%)	2.50	0.453^†^
Alcohol, 4 times/week or more	127 (13.6%)	106 (11.4%)	0.57	0.022^†^	15 (7.6%)	15 (7.6%)	1.00	1.000^†^
**Sleep and fatigue**								
Sleep problems (range 1–10)^$^	4.7 (3.0)	4.7 (3.0)	0.011	0.905^*^	3.5 (2.9)	3.8 (3.0)	0.335	0.129^*^
Fatigue (range 1–10)^$^	5.2 (3.0)	5.0 (2.9)	−0.187	0.025^*^	3.2 (2.8)	3.5 (2.8)	0.302	0.102^*^
**Depression**								
PHQ-8 (range 0–24)^$^	7.0 (5.6)	6.3 (5.2)	−0.637	**<0.001** ^*^	4.6 (4.0)	4.5 (3.8)	−0.230	0.373^*^
**HR-QoL**								
PCS (SF-12; range 0–100)^§^	40.4 (11.1)	42.3 (10.8)	1.586	**<0.001** ^*^	51.8 (7.1)	51.8 (7.5)	−0.166	0.770^*^
MCS (SF-12; range 0–100)^§^	41.4 (11.2)	42.8 (10.6)	1.539	**<0.001** ^*^	44.1 (10.2)	44.1 (9.8)	−0.151	0.832^*^
EQ-5D-5L utility values (range <0–1)^§^	0.86 (0.17)	0.88 (0.16)	0.018	**<0.001** ^*^	0.95 (0.12)	0.96 (0.08)	0.010	0.257^*^
EQ-5D-5L overall health (range 0–100)^§^	63.9 (21.2)	67.2 (20.3)	3.425	**<0.001** ^*^	81.0 (17.1)	80.7 (16.9)	−0.585	0.622^*^
**Health responsibilities**								
**Communication with healthcare providers**								
Communication with healthcare providers (range 0–5)^§^	2.11 (1.22)	2.28 (1.26)	0.172	**<0.001** ^*^	1.78 (1.33)	1.92 (1.40)	0.184	0.072^*^
**Health literacy Questionnaire**								
Find health information (range 1–4)^§^	3.03 (0.81)	3.08 (0.77)	0.084	0.008^*^	3.27 (0.77)	3.17 (0.63)	−0.109	0.150^*^
Understand information (range 1–4)^§^	3.02 (0.77)	3.10 (0.73)	0.110	**<0.001** ^*^	3.26 (0.75)	3.25 (0.64)	0.031	0.642^*^
**Healthcare utilization in the past 6 months**								
Doctor visits	4.97 (6.48)	3.61 (4.89)	−1.334	**<0.001** ^*^	2.25 (2.99)	1.54 (2.06)	−0.706	**<0.001** ^*^
A and E department visits	0.48 (2.26)	0.25 (0.76)	−0.229	**0.001** ^*^	0.21 (0.71)	0.19 (0.85)	−0.016	0.813^*^
Overnight hospital visits	0.30 (1.20)	0.18 (0.78)	−0.123	0.006^*^	0.12 (0.53)	0.09 (0.50)	−0.027	0.585^*^
Total nights in a hospital	1.13 (4.72)	0.65 (3.13)	−0.552	**<0.001** ^*^	0.25 (1.49)	0.35 (3.15)	0.099	0.738^*^
**Medication adherence**								
SMAQ (no adherence)	472 (57.8%)	454 (55.6%)	0.86	0.266^†^	33 (55.9%)	27 (45.8%)	0.50	0.238^†^
**Perceived medical errors**								
Communication doctor, %unclear	295 (35.9%)	260 (31.6%)	0.75	0.031^†^	37 (23.3%)	31 (19.5%)	0.73	0.418^†^
Perceived medical error, %yes	220 (28.7%)	150 (19.6%)	0.44	**<0.001** ^†^	28 (18.5%)	22 (14.6%)	0.63	0.327^†^
Perceived error as the problem, %yes	112 (86.2%)	102 (78.5%)	0.41	0.064^†^	12 (80.0%)	13 (86.7%)	1.50	1.000^†^

Data shown are the available data of the 1,152 participants who completed the baseline and follow-up questionnaires and attended ≥4 sessions of the CDSMP intervention, stratified by chronic condition status.

Data are mean (SD) or number of participants (%).

The effect variable shows “mean change” for continuous variables or “odds ratio” for categorical variables.

SEMCD-6, six item Self-Efficacy for Managing Chronic Disease scale; PHQ-8, Patient Health Questionnaire; HR-QoL, Health-related quality of life; PCS, Physical Component Summary of the SF-12; MCS, Mental Component Summary of the SF-12; SF-12, Short Form health survey; EQ-5D-5L, EuroQol-5 Dimensions-5 level; A and E, Accident and Emergency; SMAQ, Short Medication Adherence Questionnaire; β, beta (= unstandardized regression coefficient of the intercept).

* p-value based on linear regression; effect variable β.

† p-value based on McNemar's test; effect variable odds ratio.

§ A lower score is better.

§ A higher score is better.

# Significant p-values in bold after Bonferroni correction for multiple testing were applied (p = 0.05/26 = 0.0019).

Among caregivers without a chronic condition (*n* = 201), only a significant decrease in doctor visits was reported (1.54 vs. 2.25, *P* < 0.001). Supplementary Table 2A presents the effects of the EFFICHRONIC intervention on the total study population, and Supplementary Tables 2B–E presents the effects of the EFFICHRONIC intervention per country, educational level, sex, and age group. To determine the relevance of the statistically significant effects of the intervention, the differences in outcome scores before and after the intervention were compared with the minimal clinically important differences (MCIDs) of the respective outcome measures (39). The differences in the effect size of the depression score (PHQ-8), HR-QoL score (SF-12), health utility score (EQ-5D-5L), and overall health score (EQ-VAS) did not meet the MCIDs of these parameters (40–42). No MCIDs were available for the other parameters.

### Satisfaction and adverse events

Participants who completed the questions on experienced outcome changes at follow-up (*n* = 1,248) reported an improvement in doing at least one activity for health (85.2%), not letting health problems control their life (80.3%), ability to make decisions (56.0%), ability to express themselves (49.3%), communication with family, friends, and others (51.1%), and confidence in the healthcare system understanding their needs (45.1%) (Supplementary Table 4). The average satisfaction score with the intervention was 8.3 ± 1.7 on a scale from 0 (lowest) to 10 (highest) (Supplementary Table 4). No adverse events were reported for the intervention.

## Discussion

In this multicenter pre-post cohort study among a diverse European population, a 6-week chronic disease self-management program modestly improved self-efficacy, depression, HR-QoL, and health responsibilities in citizens with a chronic condition. However, considering these improvements, the differences shown for a part of the outcome measures (depression, HR-QoL, health utility, and overall health) were tested and found to not be clinically relevant when compared with their respective MCIDs. In addition, health behaviors did not improve.

Since the organization of healthcare in European countries varies substantially per country and, more importantly, also affects the accessibility of care (45), secondary analyses were conducted to investigate the differences between subgroups of different sociodemographic backgrounds (Supplementary Table 2B). Hardman et al. suggested a moderating effect of socioeconomic background on self-management support interventions in favor of people with high socioeconomic backgrounds (Supplementary Table 2C) (43). There is no known difference in the effect of CDSMPs between men and women; however, since mostly women participate in CDSMPs, studies might have lacked the statistical power to investigate the effect sufficiently in men (Supplementary Table 2D). Similarly, people aged 65 years and older more often finish CDSMP programs, among others participants, due to higher motivation. However, the impact of older age on the CDSMP effect remains largely unknown (Supplementary Table 2E) (44). Additional analyses show the effects per country, education level, sex, and age group; the outcome patterns of the whole study population are reflected in Supplementary Tables 2B–E, respectively; however, because the subgroups have lower numbers, there are less significant results.

Modifiable lifestyle risk factors (i.e., health behaviors) are the main drivers of chronic diseases (1), as shown in multiple well-established cohort studies, such as the Whitehall study (45), Framingham Heart Study (46), Women's Health Initiative (47), Nurses' Health Study (48), EPIC study (European Prospective Investigation into Cancer and Nutrition) (49), MESA study (Multi-Ethnic Study on Atherosclerosis) (50), and Mediators of Atherosclerosis in South Asians Living in America (MASALA) study (51). While there is a widespread belief in the importance of self-management programs for improving health behaviors in people with chronic conditions (10, 52, 53), the present study failed to show the effects of a well-established CDSMP on health behaviors. A recent study of a comparable CDSMP also reported no effect on health behaviors (54). Similarly, a meta-analysis and systematic review of self-management intervention studies showed improvement in subjective wellbeing, but overall, there were no effects on physical activity, diet and nutrition, smoking, alcohol consumption, and blood pressure (55, 56). The lack of improvement in health behaviors may be related to the intensity of the program as various systematic reviews on lifestyle interventions showed that intensive follow-up monitoring, a higher number of contact moments, face-to-face counseling, targeting multiple behaviors, and including common behavior change techniques were the most distinct factors within these interventions for changing one's health behaviors (57–60). There is no consistent evidence to substantiate that the lack of improvement could be related to the group-based community setting and/or low SES (61–63). Previous studies of the CDSMP conducted with a limited number of participants in low-SES populations showed improvements in self-efficacy, symptom management, general health, pain, and fatigue (64, 65). In contrast, interventions that target lifestyle risk factors more specifically and extensively, such as the Diabetes Prevention Program, the PREDIMED-Reus intervention, and CHIP (Complete Health Improvement Program), have proven successful in reducing the incidence of diabetes, inducing weight loss, and/or lowering blood glucose (66–68). Interestingly, a meta-analysis and a systematic review showed that behavioral treatment strategies and mitigating participation barriers improved adherence to lifestyle interventions (69, 70), which suggests that a behavior change intervention, such as the CDSMP, combined with an intervention addressing lifestyle-related risk factors, could work synergistically to reduce the burden of non-communicable chronic diseases.

Next to self-efficacy, depression, and HR-QoL, our study also showed improvement in health responsibilities (in the participants with a chronic condition): Positive changes were observed in communication with healthcare providers, perceived medical errors, healthcare utilization, and health literacy. To date, little research has been conducted on the relationship between self-management programs and health responsibilities. Previous studies have shown more benefits of self-management programs in people with low health literacy compared to those with high health literacy (71). In addition, health literacy itself may improve with a self-management program (72).

Although most self-management programs target patients with a chronic disease, some studies also demonstrated improved self-efficacy and higher HR-QoL in caregivers (73, 74). In our study, the effect of the CDSMP was less in caregivers without a chronic condition as compared with people with a chronic condition. The caregivers were more often female and unemployed; therefore, it is unclear whether this is the reason that underlies the difference in effectiveness. Alternatively, a difference in receptivity for a behavioral intervention between people with and without a chronic condition may explain this discrepancy.

### Limitations

First, a lack of a control group prevents the outcomes from being linked to the intervention since non-specific effects related to group-based intervention participation cannot be ruled out. The outcome measures were self-reported; therefore, a bias in the outcome estimates cannot be excluded and is probably less precise than objectively measured outcomes. Furthermore, the presence of selection or social desirability bias cannot be ruled out; we only have data on the sociodemographic characteristics of the participants who completed the baseline questionnaire, which makes it hard to infer the implications of our study. The intervention was targeted at a vulnerable population; however, we did not achieve in including those who are most vulnerable, for example, people who were not able to speak the local language were not able to participate. In addition, despite all efforts, many participants did not fill in one or both questionnaires and were thus excluded from analyses, resulting in a high attrition rate.

There were differences in sociodemographic variables and lifestyle factors between dropped-out participants and participants who completed the follow-up, which might have caused attrition bias. In addition, participants in the study sites were recruited in different ways. On the one hand, the heterogeneity of the study population might make it harder to infer the effects of the intervention. On the other hand, this heterogeneous study population with diverse backgrounds might enable the outcomes to reflect the general population more. As discussed above, a CDSMP intervention with a 6-week duration might have been too short for changing health behaviors since that is a complex matter, which needs lasting attention, support, and practice. Finally, the follow-up time was 6 months, which measured only “mid-term” effects.

### Future directions

It might be worthwhile to conduct a meta-analysis of chronic disease self-management studies to assess the overall effectiveness and determining factors, such as duration, content, and setting. We also recommend a longer follow-up of behavioral and/or lifestyle intervention studies to assess the durability of any observed outcomes and to include objective measures, such as blood pressure, blood glucose, and BMI. Efforts should be made to involve vulnerable groups in chronic disease management programs to address health disparities. Communities in “Blue zones,” worldwide geographical regions where people live longer and healthier lives than the average, show that active engagement with social surroundings, a sense of belonging and purpose in life, plays a vital role in chronic disease prevention (63).

## Conclusion

This CDSMP was associated with improvement in self-efficacy, depression, HR-QoL, and health responsibilities over 6 months in a diverse European population with a chronic condition. However, additional interventions targeting lifestyle risk factors are needed to improve health outcomes.

## Data availability statement

The datasets presented in this article are not readily available because, only part of the participants have given additional permission to share their pseudonymized data to other research institutes for addressing scientific questions. However, the majority of the participants have not given permission for sharing their data with other research institutes. Requests to access the datasets should be directed to h.raat@erasmusmc.nl.

## Ethics statement

The studies involving humans were approved by Ethical Committee procedures have been followed in all study sites involved. The names of the review board and the references are: Occitanie, France: The Ethics Committee of the South-west and Oversees I in Toulouse (Comité de Protection des Personnes Sud-Ouest et Outre-Mer I) approved the study on 5 November 2018; study number NCT03840447. Genoa, Italy: The Regional Ethics Committee of Liguria (Il Comitato Etico della Regione Liguria) approved the study on 27 March 2018; study number 152-2018. Rotterdam, the Netherlands: The research proposal regarding the study site in the Netherlands was reviewed by the Medical Ethics Review Committee (Medische Ethische Toetsings Commissie; METC) of the Erasmus MC University Medical Center, Rotterdam. Based on their review, the Committee concluded that the rules laid down in the Dutch Medical Research Involving Human Subjects Act (also known by the Dutch abbreviation WMO, in full Wet Medisch-wetenschappelijk Onderzoek met mensen) do not apply to this study protocol (proposal number MEC-2017-1116), and gave permission to conduct this study at Erasmus Medical Center and to submit the results for publication in a scientific journal in the future (Letter FMS/ss/MEC-2017-1116; 23 November 2017). Asturias, Spain: The Research Ethics Committee of the Principality of Asturias (Comité de Ética de la Investigación del Principado de Asturias) approved the study on 31 January 2017; study number 20/17. London, United Kingdom: The results page from the HRA decision tool of the NHS Health Research Authority states there is not a need for NHS Research Ethics Committee (NHS REC) approval for sites in England regarding this study; followed by an e-mail of the Health Research Authority, London, UK on 28 March 2019 (Queries Line REF 76/76) saying: We can confirm that as long as the project does not involve the NHS then NHS REC review and HRA Approval are not required. And: … This decision is in line with: The harmonized UK-wide edition of the Governance Arrangements for Research Ethics Committees (GAfREC) 2018; UK Policy Framework for Health and Social Care Research (2017); The National Research Ethics Service (NRES) Defining Research table and the algorithm Does my project require review by a Research Ethics Committee? … and that … it may be provided to a journal or other body as evidence if required… The studies were conducted in accordance with the local legislation and institutional requirements. The participants provided their written informed consent to participate in this study. No potentially identifiable images or data are presented in this study.

## Author contributions

HR: conceptualization, supervision, and funding acquisition. SK, ER, and HR: methodology and validation. SK: formal analysis and visualization. IF: investigation. SK, LE, and XZ: data curation. SK and ER: writing—original draft preparation. LE, IF, SST, MP-G, HR, AB, Y-MP, AP, ML-V, IDV, and XZ: writing—review and editing. IF, SST, and HR: project administration. All authors contributed to the article and approved the submitted version.
