# Supplementary material for: The effect of a community-based group intervention on chronic disease self-management in a vulnerable population
Source: Front Public Health. 2023 Aug 21;11:1221675. doi: 10.3389/fpubh.2023.1221675 (PMC10475542; doi:10.3389/fpubh.2023.1221675)
Supplement: Supplementary file 1 [file Table_1.docx]

Supplement Table 1A. Baseline characteristics of the total study population(n=1844)

|  | Study sample (n=1844) |
| --- | --- |
| Age, y | 58.6 (14.8) |
| Female sex | 1257 (69.6%) |
| Study site |  |
| the Netherlands | 388 (21.0%) |
| Italy | 331 (18.0%) |
| United Kingdom | 345 (18.7%) |
| Spain | 568 (30.8%) |
| France | 212 (11.5%) |
| Chronic disease status |  |
| Chronic condition^$^ | 1377 (81.3%) |
| No chronic condition | 316 (18.7%) |
| Current smoking | 284 (15.9%) |
| Alcohol use ≥4 times/wk | 215 (11.9%) |
| Aerobic physical activity <150 min/wk | 1103 (61.5%) |
| Fruit <3 servings/d | 1503 (83.3%) |
| Vegetables <3 servings/d | 1583 (88.0%) |
| Current depression (PHQ-8 ≥10) | 435 (26.3%) |
| Household composition |  |
| Living with others | 1221 (68.6%) |
| Living alone | 560 (31.4%) |
| Education |  |
| Primary or no education | 331 (18.7%) |
| Secondary | 1062 (59.9%) |
| Tertiary or higher | 379 (21.4%) |
| Income (net) |  |
| >€2130 per month | 558 (32.6%) |
| €1420-2130 per month | 497 (29.0%) |
| €994-1419 per month | 299 (17.5%) |
| <€993 per month | 161 (9.4%) |
| Disability or social benefit | 197 (11.5%) |
| Housing adaptation |  |
| Adapted to my needs | 1466 (83.4%) |
| Reduced accessibility | 110 (6.3%) |
| Not properly equipped | 56 (3.2%) |
| No elevator or phone | 64 (3.6%) |
| Declining or uninhabitable | 62 (3.5%) |
| Migration background | 321 (17.8%) |
| Working status |  |
| Working as employee or self-employed | 1026 (61.0%) |
| Not working | 657 (39.0%) |
| Data are mean (SD) or number of participants (%).  $ Chronic condition group includes caregivers with chronic condition  Abbreviations: PHQ-8, Patient Health Questionnaire; Net, Net income is the amount of money a person earns after taxes and deductions are taken out; SD, standard deviation | |

|  | Netherlands  (n=388) | Italy  (n=331) | United Kingdom  (n=345) | Spain  (n=568) | France  (n=212) | *p*-value |
| --- | --- | --- | --- | --- | --- | --- |
| Age, years | 56.8 (14.1) | 62.2 (13.2) | 60.7 (15.5) | 57.5 (15.8) | 55.7 (13.3) | **< 0.001*** |
| Sex, % female | 296 (77.1%) | 259 (80.4%) | 169 (49.4%) | 381 (68.5%) | 152 (75.2%) | **< 0.001**† |
| Current smoking | 31 (8.1%) | 31 (9.9%) | 20 (5.8%) | 151 (27.6%) | 51 (25.1%) | **< 0.001**† |
| Alcohol use ≥4 times/wk | 52 (13.6%) | 39 (12.1%) | 77 (22.3%) | 35 (6.4%) | 12 (5.8%) | **< 0.001**† |
| Aerobic physical activity <150 min/wk | 154 (40.2%) | 205 (64.5%) | 232 (67.2%) | 377 (69.6%) | 135 (65.9%) | **< 0.001**† |
| Fruit <3 servings/d | 342 (89.3%) | 276 (85.4%) | 285 (82.6%) | 412 (75.5%) | 188 (90.8%) | **< 0.001**† |
| Vegetables <3 servings/d | 321 (84.3%) | 293 (90.7%) | 261 (76.1%) | 515 (94.5%) | 193 (93.7%) | **< 0.001**† |
| Current depression (PHQ-8 ≥10) | 78 (21.7%) | 57 (18.9%) | 114 (34.3%) | 106 (21.9%) | 80 (44.7%) | **< 0.001**† |
| Household composition, % living alone | 156 (40.9%) | 92 (29.3%) | 79 (23.0%) | 144 (26.7%) | 89 (43.8%) | **< 0.001**† |
| Education |  |  |  |  |  | **< 0.001**† |
| Primary or no education | 17 (4.6%) | 17 (5.3%) | 19 (5.5%) | 277 (51.5%) | 1 (0.5%) |  |
| Secondary | 180 (49.2%) | 249 (77.8%) | 252 (73.0%) | 195 (36.2%) | 186 (91.6%) |  |
| Tertiary or higher | 169 (46.2%) | 54 (16.9%) | 74 (21.4%) | 66 (12.3%) | 16 (7.9%) |  |
| Income (net) |  |  |  |  |  | **< 0.001**† |
| >€2130 per month | 181 (50.8%) | 83 (26.9%) | 85 (25.3%) | 164 (32.1%) | 45 (22.4%) |  |
| €1420-2130 per month | 74 (20.8%) | 183 (59.4%) | 106 (31.5%) | 97 (19.0%) | 37 (18.4%) |  |
| €994-1419 per month | 41 (11.5%) | 23 (7.5%) | 103 (30.7%) | 90 (17.6%) | 42 (20.9%) |  |
| <€993 per month | 13 (3.7%) | 14 (4.5%) | 1 (0.3%) | 128 (25.0%) | 5 (2.5%) |  |
| Disability or social benefit | 47 (13.2%) | 5 (1.6%) | 41 (12.2%) | 32 (6.3%) | 72 (35.8%) |  |
| Housing adaptation |  |  |  |  |  | **< 0.001**† |
| Adapted to my needs | 290 (82.2%) | 295 (92.8%) | 302 (87.8%) | 443 (82.2%) | 136 (66.7%) |  |
| Reduced accessibility | 12 (3.4%) | 14 (4.4%) | 21 (6.1%) | 36 (6.7%) | 27 (13.2%) |  |
| Not properly equipped | 10 (2.8%) | 4 (1.3%) | 4 (2.1%) | 26 (4.8%) | 12 (5.9%) |  |
| No elevator or phone | 29 (8.2%) | 2 (0.6%) | 2 (0.6%) | 28 (5.2%) | 3 (1.5%) |  |
| Declining or uninhabitable | 12 (3.4%) | 3 (0.9%) | 15 (4.4%) | 6 (1.1%) | 26 (12.7%) |  |
| Migration background | 95 (24.7%) | 28 (8.8%) | 92 (26.7%) | 67 (12.2%) | 42 (20.5%) | **< 0.001**† |
| Working status, % not working | 189 (53.1%) | 174 (54.4%) | 122 (36.0%) | 130 (27.6%) | 42 (21.3%) | **< 0.001**† |
| Data are mean (SD) or number of participants (%).  Abbreviations: PHQ-8, Patient Health Questionnaire; Net, Net income is the amount of money a person earns after taxes and deductions are taken out ; SD, standard deviation.  * *p* value based on One-way analysis of variance (ANOVA); significant *p* values in bold  † *p* value based on Pearson’s chi-squared test; significant *p* values in bold | | | | | | |

## Supplement Table 1B Baseline characteristics of the study sample on country level (n=1844)

Supplement Table 2A. Effects of EFFICHRONIC intervention, in the total study population (n=1248)

| **Outcomes** | Baseline | Follow-up  (6 month) | Effect variable | Mean  change | 95% Confidence  interval | *p* value^#^ | |
| --- | --- | --- | --- | --- | --- | --- | --- |
| **Self-efficacy** |  |  |  |  |  |  | |
| SEMCD-6 (range 1-10)^§^ | 6.7 (2.1) | 7.0 (2.1) | β | 0.279 | 0.167 – 0.392 | **< 0.001*** | |
| **Health behaviors** |  |  |  |  |  |  | |
| Dietary habits |  |  |  |  |  |  | |
| Fruit <3 portions/d | 1010 (82.8%) | 999 (81.9%) | OR | 0.89 | 0.67 – 1.18 | 0.472^†^ | |
| Vegetables, <3 portions/d | 1058 (87.5%) | 1042 (86.2%) | OR | 0.80 | 0.58 – 1.11 | 0.214^†^ | |
| Physical activity |  |  |  |  |  |  | |
| Stretching/strengthening (min/wk) | 31.5 (52.3) | 32.2 (51.4) | β | 1.327 | -1.635 – 4.290 | 0.379***** | |
| Aerobic physical activity (min/wk) | 133.1 (108.1) | 136.3 (110.5) | β | 3.654 | -2.075 – 9.384 | 0.211***** | |
| Sedentary behavior (h/d) | 5.9 (2.9) | 5.7 (2.8) | β | -0.218 | -0.364 - -0.072 | 0.003***** | |
| Substance use |  |  |  |  |  |  | |
| Current smoking | 159 (13.3%) | 160 (13.4%) | OR | 1.05 | 0.57 – 1.94 | 1.000^†^ | |
| Alcohol, 4 times/wk or more | 159 (13.1%) | 135 (11.1%) | OR | 0.60 | 0.40 – 0.91 | 0.018^†^ | |
| Sleep and fatigue |  |  |  |  |  |  | |
| Sleep problems (range 1-10)^$^ | 4.4 (3.0) | 4.4 (3.0) | β | 0.038 | -0.119 – 0.195 | 0.635***** | |
| Fatigue (range 1-10)^$^ | 4.8 (3.0) | 4.7 (3.0) | β | -0.137 | -0.280 – 0.006 | 0.061* | |
| **Depression** |  |  |  |  |  |  | |
| PHQ-8 (range 0-24)^$^ | 6.4 (5.4) | 5.8 (5.0) | β | -0.583 | -0.824 - -0.342 | **< 0.001*** | |
| **HR-QoL** |  |  |  |  |  |  | |
| PCS (SF-12; range 0-100)^§^ | 43.0 (11.3) | 44.5 (10.8) | β | 1.128 | 0.646 – 1.610 | **< 0.001*** | |
| MCS (SF-12; range 0-100)^§^ | 42.0 (11.1) | 43.2 (10.4) | β | 1.280 | 0.651 – 1.909 | **< 0.001*** | |
| EQ-5D-5L utility values (range <0-1)^§^ | 0.88 (0.17) | 0.90 (0.15) | β | 0.015 | 0.008 – 0.023 | **< 0.001*** | |
| EQ-5D-5L overall health (range 0-100)^§^ | 67.9 (21.3) | 70.4 (20.3) | β | 2.581 | 1.525 – 3.638 | **< 0.001*** | |
| **Health responsibilities** |  |  |  |  |  |  | |
| Communication with healthcare providers |  |  |  |  |  |  | |
| Communication healthcare providers (range 0-5)^§^ | 2.06 (1.25) | 2.23 (1.30) | β | 0.181 | 0.109 – 0.252 | **< 0.001*** | |
| Health literacy Questionnaire |  |  |  |  |  |  | |
| Find health information (range 1-4)^§^ | 3.08 (0.80) | 3.09 (0.75) | β | 0.054 | -0.003 – 0.111 | 0.066* | |
| Understand information (range 1-4)^§^ | 3.06 (0.78) | 3.11 (0.73) | β | 0.098 | 0.040 – 0.155 | **< 0.001*** | |
| Healthcare utilization in the past 6 months |  |  |  |  |  |  | |
| Doctor visits | 4.4 (6.0) | 3.1 (4.5) | β | -1.206 | -1.513 - -0.900 | **< 0.001*** | |
| A and E department visits | 0.41 (2.02) | 0.23 (0.75) | β | -0.179 | -0.290 - -0.068 | **0.002*** | |
| Overnight hospital visits | 0.25 (1.09) | 0.15 (0.71) | β | -0.101 | -0.170 - -0.031 | 0.005***** | |
| Total nights in a hospital | 0.95 (4.28) | 0.58 (3.08) | β | -0.444 | -0.726 - -0.163 | 0.002* | |
| Medication adherence |  |  |  |  |  |  | |
| SMAQ (no adherence) | 519 (57.2%) | 497 (54.8%) | OR | 0.84 | 0.66 – 1.08 | 0.191^†^ | |
| **Perceived medical errors** |  |  |  |  |  |  | |
| Communication doctor, %unclear | 335 (33.2%) | 295 (29.2%) | OR | 0.76 | 0.60 – 0.96 | 0.022^†^ | |
| Perceived medical error, %yes | 255 (27.0%) | 174 (18.4%) | OR | 0.45 | 0.33 – 0.60 | **< 0.001**^†^ | |
| Perceived error as problem, %yes | 126 (85.7%) | 117 (79.6%) | OR | 0.53 | 0.24 – 1.13 | 0.136^†^ | |
| Data shown are the available data of the 1248 participants who completed the baseline and follow-up questionnaires and attended ≥4 sessions of the CDSMP intervention.  Data are mean (SD) or number of participants (%).  The effect variable shows ‘mean change’ for continuous variables or ‘odds ratio’ for categorical variables.  SEMCD-6, six item Self-Efficacy for Managing Chronic Disease scale; PHQ-8, Patient Health Questionnaire; HR-QoL, Health-related quality of life; PCS, Physical Component Summary of the SF-12; MCS, Mental Component Summary of the SF-12; SF-12, Short Form health survey; EQ-5D-5L, EuroQol-5 Dimensions-5 level; A and E, Accident and Emergency; SMAQ, Short Medication Adherence Questionnaire; β, beta (=unstandardized regression coefficient of the intercept). ^*^ *p* value based on linear regression; effect variable β  ^†^ *p* value based on McNemar test; effect variable odds ratio  ^$^ A lower score is better  ^§^ A higher score is better  ^#^ Significant *p* values in bold after Bonferroni correction for multiple testing was applied (P = 0.05/26 = 0.0019) | | | | | | |  |

Supplement Table 2B Effects of the EFFICHRONIC intervention by country (n=1248)

|  | Netherlands | |  | Italy | |  | United Kingdom | |  | Spain | |  | France | | |  |
| --- | --- | --- | --- | --- | --- | --- | --- | --- | --- | --- | --- | --- | --- | --- | --- | --- |
|  | (n=276) | |  | (n=214) | |  | (n=275) | |  | (n=349) | |  | (n=134) | | |  |
| **Outcomes** | Baseline | Follow-up | *p* value^#^ | Baseline | Follow-up | *p* value^#^ | Baseline | Follow-up | *p* value^#^ | Baseline | Follow-up | *p* value^#^ | Baseline | Follow-up | *p* value^#^ | |
|  |  | (6 month) |  |  | (6 month) |  |  | (6 month) |  |  | (6 month) |  |  | (6 month) |  |  |
| **Self-efficacy** |  |  |  |  |  |  |  |  |  |  |  |  |  |  |  | |
| SEMCD-6 (range 1-10)^§^ | 6.7 (1.8) | 6.8 (1.9) | 0.038* | 7.3 (1.8) | 7.3 (1.8) | 0.470* | 5.7 (2.2) | 6.4 (2.1) | **< 0.001*** | 7.6 (2.1) | 7.6 (2.1) | 0.277* | 6.0 (2.0) | 6.4 (2.1) | 0.081* | |
| **Health behaviors** |  |  |  |  |  |  |  |  |  |  |  |  |  |  |  | |
| Dietary habits |  |  |  |  |  |  |  |  |  |  |  |  |  |  |  | |
| Fruit <3 portions/d | 246 (89.5%) | 247 (89.8%) | 1.000† | 173 (83.2%) | 164 (78.8%) | 0.200† | 225 (81.8%) | 225 (81.8%) | 1.000† | 244 (73.9%) | 245 (74.2%) | 1.000† | 122 (92.4%) | 118 (89.4%) | 0.424† | |
| Vegetables, <3 portions/d | 231 (84.9%) | 222 (81.6%) | 0.175† | 183 (88.0%) | 185 (88.9%) | 0.851† | 214 (78.7%) | 206 (75.7%) | 0.291† | 308 (94.5%) | 311 (95.4%) | 0.690† | 122 (93.1%) | 118 (90.1%) | 0.424† | |
| Physical activity |  |  |  |  |  |  |  |  |  |  |  |  |  |  |  | |
| Stretching/strengthening (min/wk) | 19.9 (43.4) | 18.2 (38.9) | 0.657* | 41.7 (62.5) | 43.2 (61.3) | 0.326* | 26.4 (47.3) | 24.5 (42.8) | 0.626* | 40.6 (56.8) | 41.2 (56.4) | 0.898* | 28.2 (44.6) | 38.8 (52.8) | 0.019* | |
| Aerobic physical activity (min/wk) | 193.2 (123.4) | 193.4 (118.3) | 0.934* | 127.2 (91.5) | 133.7 (99.8) | 0.304* | 110.8 (102.9) | 112.7 (107.3) | 0.738* | 112.0 (94.3) | 108.1 (91.3) | 0.469* | 117.8 (96.6) | 144.7 (118.3) | **0.002*** | |
| Sedentary behavior (h/d) | 7.3 (2.8) | 7.0 (2.9) | 0.077* | 5.7 (2.8) | 5.6 (2.7) | 0.245* | 6.6 (3.0) | 6.3 (2.6) | 0.030* | 4.1 (2.1) | 4.2 (2.3) | 0.663* | 5.7 (2.8) | 5.3 (2.7) | 0.021* | |
| Substance use |  |  |  |  |  |  |  |  |  |  |  |  |  |  |  | |
| Current smoking | 27 (9.9%) | 26 (9.5%) | 1.000† | 21 (10.8%) | 27 (13.8%) | 0.070† | 12 (4.4%) | 10 (3.7%) | 0.727† | 71 (21.6%) | 68 (20.7%) | 0.581† | 28 (22.6%) | 29 (23.4%) | 1.000† | |
| Alcohol, 4 times/wk or more | 43 (15.6%) | 39 (14.2%) | 0.523† | 27 (13.0%) | 22 (10.6%) | 0.227† | 59 (21.5%) | 44 (16.1%) | 0.021† | 23 (7.0%) | 23 (7.0%) | 1.000† | 7 (5.3%) | 7 (5.3%) | 1.000† | |
| Sleep and fatigue |  |  |  |  |  |  |  |  |  |  |  |  |  |  |  | |
| Sleep problems (range 1-10)^$^ | 4.4 (3.0) | 4.1 (2.8) | 0.037* | 3.9 (2.9) | 4.3 (3.1) | 0.042* | 5.4 (2.9) | 5.0 (3.0) | 0.002* | 3.4 (2.9) | 4.0 (3.1) | **< 0.001*** | 5.5 (3.0) | 5.5 (3.0) | 0.586* | |
| Fatigue (range 1-10)^$^ | 5.3 (2.8) | 4.7 (2.8) | **< 0.001*** | 4.3 (2.7) | 4.4 (2.8) | 0.672* | 5.7 (2.8) | 5.4 (2.7) | 0.040* | 3.4 (3.1) | 3.7 (3.2) | 0.130* | 6.2 (2.6) | 6.0 (2.6) | 0.174* | |
| **Depression** |  |  |  |  |  |  |  |  |  |  |  |  |  |  |  | |
| PHQ-8 (range 0-24)^$^ | 5.9 (4.9) | 5.1 (4.7) | **< 0.001*** | 5.3 (4.3) | 5.4 (4.2) | 0.412* | 7.6 (6.4) | 6.7 (5.5) | 0.007* | 5.6 (5.1) | 5.4 (4.8) | 0.119* | 8.9 (5.2) | 7.4 (5.5) | 0.003* | |
| **HR-QoL** |  |  |  |  |  |  |  |  |  |  |  |  |  |  |  | |
| PCS (SF-12; range 0-100)^§^ | 46.1 (10.0) | 46.7 (9.7) | 0.133* | 48.2 (8.9) | 49.4 (8.3) | 0.017* | 37.6 (11.7) | 40.6 (11.1) | **< 0.001*** | 44.1 (10.9) | 44.4 (11.0) | 0.930* | 37.9 (10.6) | 39.8 (10.4) | 0.004* | |
| MCS (SF-12; range 0-100)^§^ | 40.6 (10.6) | 42.2 (10.3) | 0.002* | 43.6 (10.7) | 44.2 (9.6) | 0.313* | 39.8 (11.7) | 41.8 (10.3) | 0.063* | 46.4 (10.1) | 45.9 (10.4) | 0.917* | 37.2 (10.4) | 40.4 (10.7) | **0.001*** | |
| EQ-5D-5L utility values (range <0-1)^§^ | 0.95 (0.09) | 0.95 (0.08) | 0.808* | 0.95 (0.08) | 0.95 (0.07) | 0.121* | 0.86 (0.19) | 0.89 (0.16) | **< 0.001*** | 0.83 (0.18) | 0.84 (0.19) | 0.348* | 0.80 (0.20) | 0.83 (0.19) | **< 0.001*** | |
| EQ-5D-5L overall health (range 0-100)^§^ | 72.1 (17.0) | 73.4 (17.0) | 0.105* | 73.1 (19.2) | 74.1 (17.5) | 0.387* | 60.3 (23.1) | 68. 1 (22.0) | **< 0.001*** | 72.1 (21.0) | 71.3 (22.0) | 0.381* | 56.4 (21.1) | 61.0 (19.2) | 0.004* | |
| **Health responsibilities** |  |  |  |  |  |  |  |  |  |  |  |  |  |  |  | |
| Communication with healthcare providers |  |  |  |  |  |  |  |  |  |  |  |  |  |  |  | |
| Communication healthcare providers (range 0-5)^§^ | 2.5 (1.2) | 2.5 (1.3) | 0.053* | 2.0 (1.2) | 2.2 (1.3) | 0.095* | 2.2 (1.2) | 2.5 (1.1) | **< 0.001*** | 1.8 (1.3) | 1.9 (1.4) | 0.118* | 2.0 (1.2) | 2.2 (1.3) | 0.035* | |
| Health literacy Questionnaire |  |  |  |  |  |  |  |  |  |  |  |  |  |  |  | |
| Find health information (range 1-4)^§^ | 3.11 (0.69) | 3.12 (0.80) | 0.434* | 3.19 (0.75) | 3.14 (0.66) | 0.167* | 3.05 (0.82) | 3.16 (0.70) | 0.010* | 3.20 (0.74) | 3.10 (0.73) | 0.512* | 2.64 (0.94) | 2.79 (0.94) | 0.026* | |
| Understand information (range 1-4)^§^ | 2.93 (0.78) | 2.93 (0.72) | 0.575* | 3.32 (0.68) | 3.28 (0.58) | 0.629* | 2.96 (0.83) | 3.11 (0.73) | 0.008* | 3.18 (0.70) | 3.18 (0.69) | 0.138* | 2.78 (0.80) | 2.96 (0.90) | 0.007* | |
| Healthcare utilization in the past 6 months |  |  |  |  |  |  |  |  |  |  |  |  |  |  |  | |
| Doctor visits | 4.6 (6.5) | 2.8 (3.6) | **< 0.001*** | 3.8 (4.8) | 2.6 (2.9) | 0.002* | 4.8 (6.2) | 3.1 (3.2) | **< 0.001*** | 3.8 (5.5) | 2.7 (3.2) | **< 0.001*** | 5.3 (7.3) | 5.9 (9.6) | 0.503* | |
| A and E department visits | 0.13 (0.51) | 0.07 (0.30) | 0.112* | 0.18 (0.59) | 0.13 (0.41) | 0.438* | 0.67 (0.31) | 0.30 (0.73) | 0.044* | 0.60 (2.42) | 0.40 (1.13) | 0.133* | 0.33 (0.91) | 0.15 (0.56) | 0.007* | |
| Overnight hospital visits | 0.10 (0.38) | 0.09 (0.39) | 0.758* | 0.33 (1.15) | 0.13 (0.48) | 0.024* | 0.27 (1.15) | 0.13 (0.45) | 0.066* | 0.27 (0.95) | 0.18 (0.82) | 0.142* | 0.39 (1.88) | 0.28 (1.37) | 0.573* | |
| Total nights in a hospital | 0.83 (5.18) | 0.74 (3.79) | 0.139* | 1.35 (5.83) | 0.46 (1.96) | 0.039* | 0.68 (2.87) | 0.49 (2.64) | 0.269* | 0.90 (3.57) | 0.73 (3.80) | 0.622* | 1.19 (4.40) | 0.46 (2.75) | 0.063* | |
| Medication adherence |  |  |  |  |  |  |  |  |  |  |  |  |  |  |  | |
| SMAQ (no adherence) | 102 (64.6%) | 99 (62.7%) | 0.742† | 88 (59.9%) | 73 (49.7%) | 0.032† | 154 (61.8%) | 165 (66.3%) | 0.272† | 122 (48.6%) | 111 (44.2%) | 0.242† | 53 (52.0%) | 49 (48.0%) | 0.523† | |
| **Perceived medical errors** |  |  |  |  |  |  |  |  |  |  |  |  |  |  |  | |
| Communication doctor, %unclear | 29 (30.2%) | 21 (21.9%) | 0.186† | 64 (32.8%) | 58 (29.7%) | 0.496† | 113 (41.4%) | 102 (37.4%) | 0.300† | 92 (28.8%) | 73 (22.9%) | 0.046† | 37 (29.4%) | 41 (32.5%) | 0.607† | |
| Perceived medical error, %yes | 29 (29.6%) | 4 (13.8%) | **< 0.001†** | 49 (25.7%) | 50 (26.2%) | 1.000† | 67 (24.5%) | 55 (20.1%) | 0.097† | 75 (28.7%) | 35 (13.4%) | **< 0.001†** | 35 (28.9%) | 30 (24.8%) | 0.332† | |
| Perceived error as problem, %yes | 9 (81.8%) | 4 (36.4%) | 0.063† | 22 (78.6%) | 24 (85.7%) | 0.687† | 39 (90.7%) | 37 (86.0%) | 0.625† | 32 (80.0%) | 28 (70.0%) | 0.424† | 24 (96.0%) | 24 (96.0%) | 1.000† | |
| Data shown are the available data of the 1248 participants who completed the baseline and follow-up questionnaires and attended ≥4 sessions of the CDSMP intervention, stratified by country.  Data are mean (SD) or number of participants (%).  The effect variable shows ‘mean change’ for continuous variables or ‘odds ratio’ for categorical variables.  SEMCD-6, six item Self-Efficacy for Managing Chronic Disease scale; PHQ-8, Patient Health Questionnaire; HR-QoL, Health-related quality of life; PCS, Physical Component Summary of the SF-12; MCS, Mental Component Summary of the SF-12; SF-12, Short Form health survey; EQ-5D-5L, EuroQol-5 Dimensions-5 level; A and E, Accident and Emergency; SMAQ, Short Medication Adherence Questionnaire; β, beta (=unstandardized regression coefficient of the intercept). -5 level; A and E, Accident and Emergency  * p value based on linear regression; effect variable β  † p value based on McNemar test; effect variable odds ratio  $ A lower score is better  § A higher score is better  # Significant p values in bold after Bonferroni correction for multiple testing was applied (p = 0.05/26 = 0.0019) | | | | | | | | | | | | | | | | |

Supplement Table 2C Effects of the EFFICHRONIC intervention by education level (n=1213)

|  | Primary or no education | |  | Secondary | |  | Tertiary or higher | |  |
| --- | --- | --- | --- | --- | --- | --- | --- | --- | --- |
|  | (n=196) | |  | (n=741) | |  | (n=276) | |  |
| **Outcomes** | Baseline | Follow-up | *p* value^#^ | Baseline | Follow-up | *p* value^#^ | Baseline | Follow-up | *p* value^#^ |
|  |  | (6 month) |  |  | (6 month) |  |  | (6 month) |  |
| **Self-efficacy** |  |  |  |  |  |  |  |  |  |
| SEMCD-6 (range 1-10)^§^ | 7.0 (2.4) | 6.8 (2.4) | 0.858* | 6.5 (2.2) | 6.9 (2.0) | **< 0.001*** | 7.1 (1.9) | 7.3 (1.9) | 0.045* |
| **Health behaviors** |  |  |  |  |  |  |  |  |  |
| Dietary habits |  |  |  |  |  |  |  |  |  |
| Fruit <3 portions/d | 138 (73.8%) | 139 (74.3%) | 1.000† | 630 (85.6%) | 616 (83.7%) | 0.219† | 223 (81.4%) | 224 (81.8%) | 1.000† |
| Vegetables, <3 portions/d | 176 (95.1%) | 175 (94.6%) | 1.000† | 639 (87.5%) | 631 (86.4%) | 0.451† | 222 (81.9%) | 216 (79.7%) | 0.441† |
| Physical activity |  |  |  |  |  |  |  |  |  |
| Stretching/strengthening (min/wk) | 32.2 (50.7) | 31.2 (50.2) | 0.708* | 30.3 (52.3) | 31.9 (50.8) | 0.101* | 33.1 (53.1) | 34.0 (54.2) | 0.985* |
| Aerobic physical activity (min/wk) | 107.5 (94.8) | 99.5 (91.1) | 0.105* | 130.5 (108.3) | 135.6 (108.5) | 0.154* | 157.3 (113.7) | 116.2 (120.9) | 0.160* |
| Sedentary behavior (h/d) | 4.5 (2.3) | 4.4 (2.5) | 0.993* | 5.9 (2.8) | 5.6 (2.7) | 0.003* | 6.8 (3.3) | 6.6 (3.0) | 0.089* |
| Substance use |  |  |  |  |  |  |  |  |  |
| Current smoking | 31 (16.6%) | 31 (16.6%) | 1.000† | 103 (14.3%) | 108 (15.0%) | 0.442† | 21 (7.9%) | 17 (6.4%) | 0.289† |
| Alcohol, 4 times/wk or more | 15 (8.0%) | 13 (6.9%) | 0.754† | 94 (12.8%) | 78 (10.7%) | 0.064† | 48 (17.6%) | 42 (15.4%) | 0.238† |
| Sleep and fatigue |  |  |  |  |  |  |  |  |  |
| Sleep problems (range 1-10)^$^ | 3.9 (3.1) | 4.5 (3.4) | 0.049* | 4.6 (3.0) | 4.7 (3.0) | 0.684* | 4.0 (2.9) | 3.9 (2.9) | 0.848* |
| Fatigue (range 1-10)^$^ | 4.3 (3.2) | 4.6 (3.2) | 0.283* | 5.0 (3.0) | 4.8 (2.9) | 0.074* | 4.5 (2.9) | 4.3 (2.8) | 0.036* |
| **Depression** |  |  |  |  |  |  |  |  |  |
| PHQ-8 (range 0-24)^$^ | 6.6 (5.7) | 6.1 (5.2) | 0.332* | 6.8 (5.5) | 6.2 (5.1) | **< 0.001*** | 5.4 (4.6) | 4.5 (4.2) | **< 0.001*** |
| **HR-QoL** |  |  |  |  |  |  |  |  |  |
| PCS (SF-12; range 0-100)^§^ | 40.4 (11.4) | 42.0 (10.8) | 0.209* | 42.2 (11.3) | 44.1 (10.8) | **< 0.001*** | 46.6 (10.6) | 47.0 (10.2) | 0.698* |
| MCS (SF-12; range 0-100)^§^ | 44.7 (11.9) | 44.8 (11.1) | 0.871* | 41.0 (11.2) | 42.5 (10.4) | **< 0.001*** | 42.7 (10.3) | 44.2 (10.1) | 0.009* |
| EQ-5D-5L utility values (range <0-1)^§^ | 0.81 (0.20) | 0.82 (0.21) | 0.435* | 0.88 (0.16) | 0.90 (0.15) | **< 0.001*** | 0.94 (0.11) | 0.95 (0.09) | 0.027* |
| EQ-5D-5L overall health (range 0-100)^§^ | 66.0 (21.7) | 67.5 (22.3) | 0.491* | 66.0 (22.1) | 69.1 (20.6) | **< 0.001*** | 74.4 (17.5) | 76.3 (16.0) | 0.013* |
| **Health responsibilities** |  |  |  |  |  |  |  |  |  |
| Communication with healthcare providers |  |  |  |  |  |  |  |  |  |
| Communication healthcare providers (range 0-5)^§^ | 1.7 (1.2) | 1.6 (1.1) | 0.546* | 2.1 (1.2) | 2.2 (1.3) | **< 0.001*** | 2.4 (1.3) | 2.7 (1.3) | **< 0.001*** |
| Health literacy Questionnaire |  |  |  |  |  |  |  |  |  |
| Find health information (range 1-4)^§^ | 3.22 (0.70) | 3.05 (0.76) | 0.116* | 3.04 (0.85) | 3.10 (0.76) | 0.005* | 3.12 (0.68) | 3.10 (0.71) | 0.824* |
| Understand information (range 1-4)^§^ | 3.10 (0.78) | 3.08 (0.73) | 0.294* | 3.03 (0.76) | 3.14 (0.72) | **< 0.001*** | 3.09 (0.78) | 3.08 (0.72) | 1.000* |
| Healthcare utilization in the past 6 months |  |  |  |  |  |  |  |  |  |
| Doctor visits | 4.01 (6.14) | 2.72 (2.76) | 0.008* | 4.68 (6.18) | 3.32 (4.75) | **< 0.001*** | 3.71 (5.19) | 2.78 (4.31) | 0.004* |
| A and E department visits | 0.48 (1.21) | 0.36 (1.00) | 0.256* | 0.49 (2.50) | 0.23 (0.65) | 0.004* | 0.15 (0.46) | 0.14 (0.80) | 0.827* |
| Overnight hospital visits | 0.21 (0.73) | 0.26 (1.03) | 0.601* | 0.33 (1.32) | 0.16 (0.73) | 0.003* | 0.10 (0.39) | 0.05 (0.27) | 0.064* |
| Total nights in a hospital | 0.95 (5.36) | 0.74 (3.98) | 0.666* | 1.12 (4.47) | 0.67 (3.24) | 0.003* | 0.30 (1.54) | 0.11 (0.57) | 0.043* |
| Medication adherence |  |  |  |  |  |  |  |  |  |
| SMAQ (no adherence) | 76 (46.3%) | 72 (43.9%) | 0.683† | 321 (58.3%) | 309 (56.1%) | 0.372† | 115 (65.0%) | 108 (61.0%) | 0.392† |
| **Perceived medical errors** |  |  |  |  |  |  |  |  |  |
| Communication doctor, %unclear | 52 (29.2%) | 38 (21.3%) | 0.070† | 211 (34.0%) | 205 (33.0%) | 0.711† | 64 (33.5%) | 47 (24.6%) | 0.027† |
| Perceived medical error, %yes | 23 (16.7%) | 16 (11.6%) | 0.265† | 170 (28.5%) | 128 (21.4%) | **< 0.001†** | 56 (29.5%) | 28 (14.7%) | **< 0.001†** |
| Perceived error as problem, %yes | ε | ε | ε | ε | ε | ε | ε | ε | ε |
| Data shown are the available data of the 1248 participants who completed the baseline and follow-up questionnaires and attended ≥4 sessions of the CDSMP intervention, stratified by education level.  Data are mean (SD) or number of participants (%).  The effect variable shows ‘mean change’ for continuous variables or ‘odds ratio’ for categorical variables.  SEMCD-6, six item Self-Efficacy for Managing Chronic Disease scale; PHQ-8, Patient Health Questionnaire; HR-QoL, Health-related quality of life; PCS, Physical Component Summary of the SF-12; MCS, Mental Component Summary of the SF-12; SF-12, Short Form health survey; EQ-5D-5L, EuroQol-5 Dimensions-5 level; A and E, Accident and Emergency; SMAQ, Short Medication Adherence Questionnaire; β, beta (=unstandardized regression coefficient of the intercept).  * p value based on linear regression; effect variable β  † p value based on McNemar test; effect variable odds ratio  $ A lower score is better  § A higher score is better  # Significant p values in bold after Bonferroni correction for multiple testing was applied (p = 0.05/26 = 0.0019)  ε Numbers too low for statistical test. | | | | | | | | | |

Supplement Table 2D Effects of the EFFICHRONIC intervention by sex (n=1228)

|  | Male | |  | Female | |  |
| --- | --- | --- | --- | --- | --- | --- |
|  | (n=394) | |  | (n=834) | |  |
| **Outcomes** | Baseline | Follow-up | *p* value^#^ | Baseline | Follow-up | *p* value^#^ |
|  |  | (6 month) |  |  | (6 month) |  |
| **Self-efficacy** |  |  |  |  |  |  |
| SEMCD-6 (range 1-10)^§^ | 6.6 (2.2) | 6.9 (2.3) | **< 0.001*** | 6.8 (2.1) | 7.0 (2.1) | 0.005* |
| **Health behaviors** |  |  |  |  |  |  |
| Dietary habits |  |  |  |  |  |  |
| Fruit <3 portions/d | 336 (86.6%) | 340 (87.6%) | 0.678 | 665 (81.0%) | 649 (79.0%) | 0.205 |
| Vegetables, <3 portions/d | 339 (88.1%) | 344 (89.4%) | 0.522 | 710 (87.3%) | 689 (84.7%) | 0.050 |
| Physical activity |  |  |  |  |  |  |
| Stretching/strengthening (min/wk) | 40.6 (59.6) | 39.1 (56.1) | 0.816* | 27.3 (48.0) | 28.6 (48.3) | 0.321* |
| Aerobic physical activity (min/wk) | 140.0 (112.8) | 142.3 (117.2) | 0.579* | 130.2 (106.0) | 134.4 (107.3) | 0.225* |
| Sedentary behavior (h/d) | 6.2 (3.1) | 6.0 (2.9) | 0.175* | 5.7 (2.9) | 5.5 (2.8) | 0.006* |
| Substance use |  |  |  |  |  |  |
| Current smoking | 64 (16.6%) | 62 (16.1%) | 0.804 | 92 (11.5%) | 95 (11.9%) | 0.690 |
| Alcohol, 4 times/wk or more | 83 (21.4%) | 72 (18.6%) | 0.152 | 74 (9.1%) | 62 (7.6%) | 0.104 |
| Sleep and fatigue |  |  |  |  |  |  |
| Sleep problems (range 1-10)^$^ | 4.1 (3.0) | 4.2 (3.0) | 0.388* | 4.6 (3.0) | 4.6 (3.1) | 0.971* |
| Fatigue (range 1-10)^$^ | 4.4 (3.1) | 4.3 (2.9) | 0.285* | 5.0 (3.0) | 4.9 (3.0) | 0.089* |
| **Depression** |  |  |  |  |  |  |
| PHQ-8 (range 0-24)^$^ | 6.0 (5.4) | 5.6 (5.2) | 0.035* | 6.6 (5.4) | 5.9 (4.9) | **< 0.001*** |
| **HR-QoL** |  |  |  |  |  |  |
| PCS (SF-12; range 0-100)^§^ | 42.3 (11.7) | 44.1 (11.0) | 0.009* | 43.3 (11.1) | 44.6 (10.7) | **< 0.001*** |
| MCS (SF-12; range 0-100)^§^ | 43.0 (11.1) | 44.2 (9.9) | 0.023* | 41.5 (11.1) | 42.8 (10.7) | **0.001*** |
| EQ-5D-5L utility values (range <0-1)^§^ | 0.88 (0.17) | 0.90 (0.15) | 0.006* | 0.88 (0.17) | 0.90 (0.15) | **0.001*** |
| EQ-5D-5L overall health (range 0-100)^§^ | 67.9 (20.6) | 71.1 (19.4) | **< 0.001*** | 67.8 (21.7) | 70.2 (20.6) | **< 0.001*** |
| **Health responsibilities** |  |  |  |  |  |  |
| Communication with healthcare providers |  |  |  |  |  |  |
| Communication healthcare providers (range 0-5)^§^ | 2.0 (1.3) | 2.2 (1.3) | 0.066* | 2.1 (1.2) | 2.3 (1.3) | **< 0.001*** |
| Health literacy Questionnaire |  |  |  |  |  |  |
| Find health information (range 1-4)^§^ | 3.12 (0.78) | 3.13 (0.72) | 0.260* | 3.05 (0.81) | 3.07 (0.77) | 0.176* |
| Understand information (range 1-4)^§^ | 3.12 (0.75) | 3.14 (0.69) | 0.122* | 3.02 (0.79) | 3.10 (0.75) | 0.005* |
| Healthcare utilization in the past 6 months |  |  |  |  |  |  |
| Doctor visits | 4.69 (6.48) | 3.30 (5.34) | **< 0.001*** | 4.26 (5.81) | 3.08 (4.06) | **< 0.001*** |
| A and E department visits | 0.55 (2.25) | 0.25 (0.90) | 0.009* | 0.35 (1.91) | 0.22 (0.68) | 0.056* |
| Overnight hospital visits | 0.31 (0.92) | 0.14 (0.53) | **< 0.001*** | 0.23 (1.16) | 0.14 (0.63) | 0.050* |
| Total nights in a hospital | 1.48 (5.66) | 0.74 (3.55) | 0.003* | 0.70 (3.40) | 0.51 (2.85) | 0.149* |
| Medication adherence |  |  |  |  |  |  |
| SMAQ (no adherence) | 175 (55.6%) | 162 (51.4%) | 0.232† | 341 (58.2%) | 330 (56.3%) | 0.422† |
| **Perceived medical errors** |  |  |  |  |  |  |
| Communication doctor, %unclear | 118 (34.8%) | 106 (31.3%) | 0.266† | 215 (32.5%) | 188 (28.4%) | 0.060† |
| Perceived medical error, %yes | 83 (25.2%) | 65 (19.8%) | 0.036† | 171 (28.2%) | 107 (17.6%) | **< 0.001†** |
| Perceived error as problem, %yes | 49 (89.1%) | 43 (78.2%) | 0.109† | 76 (83.5%) | 73 (80.2%) | 0.648† |
| Data shown are the available data of the 1248 participants who completed the baseline and follow-up questionnaires and attended ≥4 sessions of the CDSMP intervention, stratified by sex. 1 participant had sex 'other', they were left out of this analysis.  Data are mean (SD) or number of participants (%).  The effect variable shows ‘mean change’ for continuous variables or ‘odds ratio’ for categorical variables.  SEMCD-6, six item Self-Efficacy for Managing Chronic Disease scale; PHQ-8, Patient Health Questionnaire; HR-QoL, Health-related quality of life; PCS, Physical Component Summary of the SF-12; MCS, Mental Component Summary of the SF-12; SF-12, Short Form health survey; EQ-5D-5L, EuroQol-5 Dimensions-5 level; A and E, Accident and Emergency; SMAQ, Short Medication Adherence Questionnaire; β, beta (=unstandardized regression coefficient of the intercept).  * p value based on linear regression; effect variable β  † p value based on McNemar test; effect variable odds ratio  $ A lower score is better  § A higher score is better  # Significant p values in bold after Bonferroni correction for multiple testing was applied (p = 0.05/26 = 0.0019) | | | | | | |

Supplement Table 2E Effects of the EFFICHRONIC intervention by age group (n=1226)

|  | Age <65 years | |  | Age ≥ 65 years | |  |
| --- | --- | --- | --- | --- | --- | --- |
|  | (n=732) | |  | (n=494) | |  |
| **Outcomes** | Baseline | Follow-up | *p* value^#^ | Baseline | Follow-up | *p* value^#^ |
|  |  | (6 month) |  |  | (6 month) |  |
| **Self-efficacy** |  |  |  |  |  |  |
| SEMCD-6 (range 1-10)^§^ | 6.6 (2.2) | 7.0 (2.1) | **< 0.001*** | 6.9 (2.1) | 7.0 (2.1) | 0.191* |
| **Health behaviors** |  |  |  |  |  |  |
| Dietary habits |  |  |  |  |  |  |
| Fruit <3 portions/d | 620 (85.6%) | 616 (85.1%) | 0.749† | 378 (78.6%) | 371 (77.1%) | 0.551† |
| Vegetables, <3 portions/d | 620 (86.4%) | 622 (86.6%) | 0.913† | 426 (89.3%) | 409 (85.7%) | 0.036† |
| Physical activity |  |  |  |  |  |  |
| Stretching/strengthening (min/wk) | 32.4 (55.2) | 34.4 (54.6) | 0.319* | 30.1 (47.3) | 28.7 (45.7) | 0.928* |
| Aerobic physical activity (min/wk) | 137.4 (118.1) | 143.5 (117.9) | 0.131* | 127.7 (90.4) | 126.7 (94.9) | 0.919* |
| Sedentary behavior (h/d) | 6.1 (3.0) | 5.8 (2.9) | **0.001*** | 5.4 (2.7) | 5.4 (2.6) | 0.660* |
| Substance use |  |  |  |  |  |  |
| Current smoking | 146 (20.5%) | 149 (21.0%) | 0.736† | 13 (2.8%) | 11 (2.4%) | 0.687† |
| Alcohol, 4 times/wk or more | 61 (8.5%) | 57 (7.9%) | 0.678† | 96 (20.0%) | 77 (16.0%) | 0.005† |
| Sleep and fatigue |  |  |  |  |  |  |
| Sleep problems (range 1-10)^$^ | 4.5 (3.1) | 4.4 (3.1) | 0.400* | 4.2 (2.9) | 4.4 (3.0) | 0.078* |
| Fatigue (range 1-10)^$^ | 5.0 (3.1) | 4.7 (3.0) | **< 0.001*** | 4.4 (2.9) | 4.6 (2.9) | 0.149* |
| **Depression** |  |  |  |  |  |  |
| PHQ-8 (range 0-24)^$^ | 7.3 (5.6) | 6.3 (5.1) | **< 0.001*** | 5.1 (4.8) | 5.1 (4.6) | 0.890* |
| **HR-QoL** |  |  |  |  |  |  |
| PCS (SF-12; range 0-100)^§^ | 44.2 (11.6) | 46.2 (10.8) | **< 0.001*** | 41.2 (11.6) | 41.9 (10.3) | 0.069* |
| MCS (SF-12; range 0-100)^§^ | 40.1 (11.1) | 42.4 (10.3) | **< 0.001*** | 45.0 (10.4) | 44.7 (10.3) | 0.981* |
| EQ-5D-5L utility values (range <0-1)^§^ | 0.88 (0.17) | 0.90 (0.15) | **< 0.001*** | 0.88 (0.17) | 0.89 (0.16) | 0.514* |
| EQ-5D-5L overall health (range 0-100)^§^ | 67.2 (22.2) | 71.0 (20.6) | **< 0.001*** | 69.1 (19.7) | 69.7 (19.5) | 0.380* |
| **Health responsibilities** |  |  |  |  |  |  |
| Communication with healthcare providers |  |  |  |  |  |  |
| Communication healthcare providers (range 0-5)^§^ | 2.05 (1.26) | 2.25 (1.32) | **< 0.001*** | 2.07 (1.23) | 2.19 (1.28) | 0.037* |
| Health literacy Questionnaire |  |  |  |  |  |  |
| Find health information (range 1-4)^§^ | 3.06 (0.85) | 3.08 (0.77) | 0.091* | 3.09 (0.73) | 3.14 (0.71) | 0.215* |
| Understand information (range 1-4)^§^ | 3.04 (0.81) | 3.10 (0.74) | 0.002* | 3.10 (0.73) | 3.14 (0.69) | 0.158 |
| Healthcare utilization in the past 6 months |  |  |  |  |  |  |
| Doctor visits | 4.44 (6.28) | 3.16 (5.15) | **< 0.001*** | 4.29 (5.56) | 3.16 (3.35) | **< 0.001*** |
| A and E department visits | 0.46 (2.54) | 0.21 (0.82) | 0.006* | 0.34 (0.83) | 0.27 (0.65) | 0.075* |
| Overnight hospital visits | 0.26 (1.11) | 0.13 (0.70) | 0.007* | 0.25 (1.08) | 0.19 (0.75) | 0.286* |
| Total nights in a hospital | 1.06 (4.61) | 0.45 (2.65) | 0.001* | 0.82 (3.80) | 0.79 (3.64) | 0.469* |
| Medication adherence |  |  |  |  |  |  |
| SMAQ (no adherence) | 311 (65.2%) | 297 (62.3%) | 0.254† | 201 (48.0%) | 193 (46.1%) | 0.536† |
| **Perceived medical errors** |  |  |  |  |  |  |
| Communication doctor, %unclear | 210 (36.3%) | 197 (34.1%) | 0.356† | 124 (29.6%) | 96 (22.9%) | 0.013† |
| Perceived medical error, %yes | 178 (32.4%) | 117 (21.3%) | **< 0.001†** | 75 (19.6%) | 54 (14.1%) | 0.015† |
| Perceived error as problem, %yes | 96 (91.4%) | 88 (83.8%) | 0.134† | 30 (73.2%) | 26 (70.7%) | 1.000† |
| Data shown are the available data of the 1248 participants who completed the baseline and follow-up questionnaires and attended ≥4 sessions of the CDSMP intervention, stratified by age.  Data are mean (SD) or number of participants (%).  The effect variable shows ‘mean change’ for continuous variables or ‘odds ratio’ for categorical variables.  SEMCD-6, six item Self-Efficacy for Managing Chronic Disease scale; PHQ-8, Patient Health Questionnaire; HR-QoL, Health-related quality of life; PCS, Physical Component Summary of the SF-12; MCS, Mental Component Summary of the SF-12; SF-12, Short Form health survey; EQ-5D-5L, EuroQol-5 Dimensions-5 level; A and E, Accident and Emergency; SMAQ, Short Medication Adherence Questionnaire; β, beta (=unstandardized regression coefficient of the intercept).  * p value based on linear regression; effect variable β  † p value based on McNemar test; effect variable odds ratio  $ A lower score is better  § A higher score is better  # Significant p values in bold after Bonferroni correction for multiple testing was applied (p = 0.05/26 = 0.0019) | | | | | | |

Supplementary Table 3 Included versus excluded participants

|  | Included  (n=1152) | Excluded  (n=692) | *p* value^∩^ |
| --- | --- | --- | --- |
| Age, y | 59.8 (14.2) | 56.4 (15.7) | **0.004** |
| Female sex | 765 (67.1%) | 492 (73.9%) | **0.008** |
| Study site |  |  | **< 0.001** |
| the Netherlands | 194 (16.8%) | 194 (28.0%) |  |
| Italy | 214 (18.6%) | 117 (16.9%) |  |
| United Kingdom | 275 (23.9%) | 70 (10.1%) |  |
| Spain | 336 (29.2%) | 232 (33.5%) |  |
| France | 133 (11.5%) | 79 (11.4%) |  |
| Type of participant |  |  | 0.157 |
| Citizen with chronic condition | 843 (73.2%) | 381 (70.4%) |  |
| Caregiver | 201 (17.4%) | 115 (21.3%) |  |
| Caregiver with chronic condition | 108 (9.4%) | 45 (8.3%) |  |
| Current smoking | 158 (13.9%) | 126 (19.3%) | **0.003** |
| Alcohol use ≥4 times/wk | 143 (12.5%) | 72 (11.0%) | 0.328 |
| Aerobic physical activity <150 min/wk | 128.3 (104.3) | 129.6 (117.7) | **0.031** |
| Fruit <3 servings/d | 943 (82.4%) | 560 (85.0%) | 0.151 |
| Vegetables <3 servings/d | 995 (87.4%) | 588 (89.2%) | 0.239 |
| Current depression (PHQ-8 ≥10) | 274 (25.9%) | 161 (27.0%) | 0.627 |
| Household composition, living alone | 342 (30.3%) | 218 (33.3%) | 0.191 |
| Education |  |  | **0.014** |
| Primary or no education | 192 (17.0%) | 139 (21.6%) |  |
| Secondary | 704 (62.4%) | 358 (55.7%) |  |
| Tertiary or higher | 233 (20.6%) | 146 (22.7%) |  |
| Income (net) |  |  | **0.001** |
| >€2130 per month | 347 (31.7%) | 211 (34.2%) |  |
| €1420-2130 per month | 348 (31.8%) | 149 (24.1%) |  |
| €994-1419 per month | 196 (17.9%) | 103 (16.7%) |  |
| <€993 per month | 87 (7.9%) | 74 (12.0%) |  |
| Disability or social benefit | 117 (10.7%) | 80 (13.0%) |  |
| Housing adaptation |  |  | **0.004** |
| Adapted to my needs | 950 (84.4%) | 516 (81.5%) |  |
| Reduced accessibility | 80 (7.1%) | 30 (4.7%) |  |
| Not properly equipped | 31 (2.8%) | 25 (3.9%) |  |
| No elevator or phone | 33 (2.9%) | 31 (4.9%) |  |
| Declining or uninhabitable | 31 (2.8%) | 31 (4.9%) |  |
| Migration background | 206 (18.1%) | 118 (17.9%) | 0.937 |
| Working status, not working | 408 (37.9%) | 249 (41.0%) | 0.210 |
| Data are mean (SD) or number of participants (%).  Abbreviations: PHQ-8, Patient Health Questionnaire; Net: Net income is the amount of money a person earns after taxes and deductions are taken out; SD, standard deviation  ∩ *p* = 0.05, significant *p* values in bold | | | |

Supplementary Table 4 Participants satisfaction and experienced changes after attending the EFFICHRONIC project (n=1248)

|  |  |
| --- | --- |
| Doing at least one activity for health |  |
| Strongly disagree /Disagree /Neither agree nor disagree | 181 (14.8%) |
| Strongly agree /Agree | 1045 (85.2%) |
| Not letting health problems control life |  |
| Strongly disagree /Disagree /Neither agree nor disagree | 236 (19.7%) |
| Strongly agree /Agree | 961 (80.3%) |
| Ability to make decisions |  |
| Not at all / A little bit / Moderately | 536 (44.0%) |
| Quite a bit / Extremely | 681 (56.0%) |
| Ability to express themselves |  |
| Not at all / A little bit / Moderately | 608 (50.7%) |
| Quite a bit / Extremely | 592 (49.3%) |
| Communication with family, friends and others |  |
| Not at all / A little bit / Moderately | 584 (48.9%) |
| Quite a bit / Extremely | 610 (51.1%) |
| Confidence in the health system |  |
| Not at all / A little bit / Moderately | 656 (54.9%) |
| Quite a bit / Extremely | 538 (45.1%) |
| Satisfaction score (0-10) | 8.3 (1.7) |
| Data are mean (SD) or number of participants (%). | |
